# Factors associated to chronic kidney disease in people living with HIV/AIDS*

**DOI:** 10.1590/1518-8345.3553.3331

**Published:** 2020-07-15

**Authors:** Priscila Silva Pontes, Antonio Ruffino-Netto, Luciana Kusumota, Christefany Régia Braz Costa, Elucir Gir, Renata Karina Reis

**Affiliations:** 1 Universidade de São Paulo, Escola de Enfermagem de Ribeirão Preto, PAHO/WHO Collaborating Centre at the Nursing Research Development, Ribeirão Preto, SP, Brazil.; 2 Universidade de São Paulo, Faculdade de Medicina de Ribeirão Preto, Departamento de Medicinal Social, Ribeirão Preto, SP, Brazil.

**Keywords:** HIV, Renal Insufficiency, Chronic, Glomerular Filtration Rate, Antiretroviral Therapy, Highly Active, Risk Factors, Comorbidity, HIV, Insuficiência Renal Crônica, Taxa de Filtração Glomerular, Terapia Antirretoviral de Alta Atividade, Fatores de Risco, Comorbidade, VIH, Insuficiencia Renal Crónica, Tasa de Filtración Glomerular, Terapia Antirretroviral Altamente Activa, Factores de Riesgo, Comorbilidad

## Abstract

**Objective:**

to analyze the factors associated to chronic kidney disease in people living with HIV (PLHIV).

**Method:**

a paired case-control study (4 controls for each case) carried out in a specialized care service in the Southeastern of Brazil, by analyzing PLHIV medical records. The sample consisted of 85 participants, corresponding to 17 cases and 68 controls. Pearson’s chi-square test (Χ^2^) and Fisher’s exact test, logistic regression, Odds Ratio (OR), 95% Confidence Interval (CI) and p<0.05 were used. SPSS version 25.0 and R Core Team, 2018 version 3.5.1 were used.

**Results:**

the factors associated with chronic kidney disease identified in this study were the following: presence of Systemic Arterial Hypertension [OR=5.8, CI (95%)=1.84-18.42, p=0.001] and use of nephrotoxic anti-retrovirals in the previous therapeutic regimen [OR=3.3, CI (95%)=1.105-10.221, p=0.028]. On the other hand, age below 40 years old [OR: 0.122, CI (95%)=0.015-0.981, p=0.022] was identified as a protective factor.

**Conclusion:**

the PLHIV under study have multi-factorial exposure associated with chronic kidney disease. However, knowing these factors helps to identify the existing risks and/or renal dysfunction, in addition to supporting the clinical decision of the health professionals who directly assist them.

## Abstract (pt)

**Objetivo:**

analisar os fatores associados à doença renal crônica em pessoas vivendo com HIV (PVHIV).

**Método:**

estudo do tipo caso-controle pareado (4 controles para cada caso), realizado em um serviço de atendimento especializado no sudeste do Brasil, por meio de análise de prontuários de PVHIV. A amostra foi de 85 participantes, correspondendo a 17 casos e 68 controles. Utilizou-se teste qui-quadrado de Pearson (Χ^2^) e teste exato de Fisher, regressão logística, *Odds Ratio* (OR), Intervalo de Confiança (IC) de 95% e p<0,05. Utilizou-se os programas SPSS versão 25.0 e R Core Team, 2018 versão 3.5.1.

**Resultados:**

os fatores associados à doença renal crônica identificados neste estudo foram: a presença de Hipertensão Arterial Sistêmica [OR=5,8, IC (95%)=1,84-18,42, p=0,001] e o uso dos antirretrovirais nefrotóxicos em esquema terapêutico anterior [OR=3,3, IC (95%)=1,105-10,221, p=0,028]. Por outro lado, a idade menor de 40 anos [OR: 0,122, IC (95%)= 0,015-0,981, p=0,022] foi identificada como um fator de proteção.

**Conclusão:**

as PVHIV estudadas possuem exposição multifatorial associadas à doença renal crônica. Entretanto, conhecer esses fatores auxilia na identificação dos riscos e/ou disfunção renal existentes, além de dar suporte na decisão clínica dos profissionais da saúde que os assistem diretamente.

## Abstract (es)

**Objetivo:**

analizar los factores asociados a la enfermedad renal crónica en personas que viven con VIH/SIDA (PVVIH).

**Método:**

estudio del tipo caso-control emparejado (4 controles para cada caso), realizado en un servicio de atención especializado en el sudeste de Brasil por medio del análisis de registros médicos de PVVIH. La muestra estuvo compuesta por 85 participantes, correspondiendo a 17 casos y 68 controles. Se utilizó la prueba de chi cuadrado de Pearson (Χ^2^) y la prueba exacta de Fisher, regresión logística, *Odds*Ratio (OR), Intervalo de Confianza (IC) del 95%, y p<0,05. Se utilizaron los programas SPSS versión 25.0 y R Core Team, 2018 versión 3.5.1.

**Resultados:**

los factores asociados a la enfermedad renal crónica identificados en este estudio fueron los siguientes: presencia de Hipertensión Arterial Sistémica [OR=5,8, IC (95%)=1,84-18,42, p=0,001] y uso de antirretrovirales nefrotóxicos en esquema terapéutico anterior [OR=3,3, IC (95%)=1,105-10,221, p=0,028]. Por otra parte, la edad menor que 40 años [OR: 0,122, IC (95%)=0,015-0,981, p=0,022] se identificó como un factor de protección.

**Conclusión:**

las PVVIH estudiadas presentan exposición multifactorial asociada a la enfermedad renal crónica. Sin embargo, conocer estos factores ayuda a identificar los riesgos y/o la disfunción renal existentes, además de servir como soporte para la decisión clínica de los profesionales de la salud que atienden directamente a estas personas.

## Introduction

In the last three decades, the occurrence of AIDS is considered one of the greatest challenges for public health worldwide. AIDS is the most important and devastating contemporary epidemic^(^ 1 ^)^.

According to the most recent world report, until the year 2017, there were about 36.9 million people living with HIV/AIDS (PLHIV), of which 75% were aware of their serological status^(^ 2 ^)^. Among those who have already been diagnosed, four out of five are using Anti-retroviral Therapies (ARTs) (79%). Among those who have already had access to the treatment, 81% had an undetectable viral load^(^ 2 ^)^.

Scientific and technological developments in the therapeutic field allowed for the advent of ART, enabling a better prognosis and, consequently, a reduction in morbidity and mortality^(^ 3 ^)^. After the success of combinations of anti-retroviral drugs (ARDs) to control viral replication, PLHIV have achieved better quality of life and greater longevity^(^ 4 ^-^ 5 ^)^. This scenario, therefore, attributes to the HIV infection a chronic condition of the disease^(^ 5 ^)^.

However, PLHIV started to present high risks of developing comorbidities (cardiovascular, liver and kidney diseases), as a consequence of the complex association between immunodeficiency, chronic inflammation, aging, and toxicity of anti-retrovirals, expanding the focus of care for a new profile of PLHIV^(^ 6 ^-^ 7 ^)^.

Faced with the new conjuncture of the health of PLHIV, previous studies have signaled a concern with chronic diseases, among them, chronic kidney disease (CKD)^(^ 7 ^-^ 8 ^)^. The diverse causes and pathogenic mechanisms of kidney diseases found in these people are complex, multi-factorial and have as an aggravating factor the inflammatory processes resulting from HIV infection and the cumulative exposure of potentially nephrotoxic anti-retrovirals^(^ 8 ^-^ 10 ^)^.

CKD in PLHIV, therefore, presents an increasing and higher form in comparison to the general population^(^ 7 ^,^ 11 ^-^ 12 ^)^, which represents clinical, managerial and economic challenges for the health care of these individuals^(^ 13 ^)^.

A number of studies reveal a higher prevalence of CKD in PLHIV when compared to people not infected by the virus^(^ 11 ^,^ 14 ^-^ 15 ^)^, presenting an up to 20 times greater risk of progressing to Terminal Chronic Kidney Disease (TCKD)^(^ 15 ^)^.

The prevalence of CKD associated with HIV varies geographically, by population and by period of the year^(^ 13 ^,^ 16 ^-^ 17 ^)^, due to genetic heterogeneity, to the initiation of the ART^(^ 18 ^)^ and to the different definition methods for CKD in each region, varying between 2% and 38%^(^ 13 ^,^ 19 ^)^. A study carried out in the Southeast region of Brazil showed that 35% of PLHIV using nephrotoxic ARDs for at least 6 months showed changes in the renal function^(^ 20 ^)^.

Therefore, the high number of PLHIV with CKD portrays an issue to be addressed by the health systems in developing countries that have a high prevalence of HIV and where access to CKD care is insufficient^(^ 13 ^)^. This increase in the number of cases leads to an increase in the use of the health resources and, consequently, to an increase in health care assistance costs^(^ 13 ^)^.

Due to the relevance of the matter^(^ 11 ^-^ 12 ^)^ and to the scarcity of research studies on this topic at the national level^(^ 20 ^)^, it is necessary to deepen studies on it and to expand the vision of comprehensive care for PLHIV, with the aim of preventing diseases, promoting health and timely treatment^(^ 20 ^)^. In view of the above, the objective of this study is to analyze the factors associated with CKD in PLHIV.

## Method

In this study, a case-control design was analyzed, which was initially nested in a descriptive-analytical, retrospective study, performed on clinical records of PLHIV using ART. The study took place in one of the five Specialized Care Services (*Serviços de Atendimento Especializado*, SAE) for PLHIV in a city in the inland of São Paulo, from February to June 2018. This SAE serves the majority of the population of the municipality, including other surrounding municipalities.

From the eligible population of 776 PLHIV cared for in the SAE in the year 2017, the sample was calculated, necessary for the initial phase of the study. For the calculation of the sample size, the following formula: $n=\frac{Z\alpha^{2}.\left(P.Q\right)}{d^{2}}$, where *n* is the sample size, *Z* is the variable reduced to a , , and a level of accuracy of . Resulting in an of 384. As it is a finite population, that is, $\frac{n}{N}=\frac{384}{776}=0,49$, is greater than 0.05. Therefore, a correction of the sample size was made using the following formula: $n_{c}=\frac{n}{1+\frac{n-1}{N}}$, being the corrected sample size, which resulted in a sample size of 258.

Initially, a sample of 258 participants was analyzed for the descriptive-analytical design. The subjects were successively included in the study, as the patients’ medical records were located in the service files. From this first analysis, the first medical record that met the inclusion and exclusion criteria was selected. In this process, 543 records were analyzed, 285 of which were excluded by the exclusion criterion.

After the analysis of the 258 participants, the following were selected as cases: PLHIV who had at least two measures of the estimate Glomerular Filtration Rate (eGFR) with an interval of 3 months or more than one eGFR<60 mL/min/1.73 m2 estimated by the Chronic Kidney Disease Epidemiology Collaboration (CKD-EPI)(21) group. The criterion for selecting the controls was for convenience, that is, the first medical record that met the inclusion criteria was selected (that is, after the identification of the cases, the first four medical records were selected immediately after the registration of the cases).

The following were considered as inclusion criteria: PLHIV registered and undergoing clinical-outpatient follow-up in the SAE chosen for the study; of both genders, over 18 years old; using ART for more than 6 months; having medical records and more than one creatinine test result available; the following were the exclusion criteria: individuals diagnosed with acute or chronic kidney disease prior to the diagnosis of HIV positive; being on hemodialysis before starting ART, and having abandoned the use of anti-retrovirals.

For this case-control study, the pairing ratio chosen was 1:4, that is, for each case, four controls were chosen. This proportion, according to Miettnen (1976), increases the degree of similarity between cases and controls(22). After a careful selection of laboratory tests, according to the criteria described, from the 258 medical records 17 cases and 68 controls were identified. In the present study, it was considered convenient not to use any variable to be paired and, for that reason, all the variables were collected and analyzed as exposure variables.

The data related to sociodemographic, behavioral, clinical and laboratory variables found in the medical records of the research participants were collected by the researcher herself using an instrument built for this study and submitted to face validation and content examination by three specialized professionals (two infectologists and a nephrologist). The data collection instrument, the Free and Informed Consent Form, and the validation instrument of the collection instrument were sent by e-mail with a period of 15 days to return the assessment. The researchers evaluated the instrument in terms of acceptance of the questioning, easy understanding, relevance of the items, clarity in the wording, presence of ambiguities, and were able to make suggestions for changes. After the analysis of the judges and necessary adjustments, a pilot study was carried out with 15 records of PLHIV in this same SAE.

Regarding the variables considered as specific associated factors for the development of CKD, the following data were collected: gender (male/female), age (>40 years old/<40 years old), skin color (white, brown, black, yellow, undeclared ), smoking (yes/no), personal history (PH) of Systemic Arterial Hypertension (SAH) (yes/no), Diabetes Mellitus (DM) (yes/no), cardiovascular disease (CVD) (yes/no), dyslipidemia (DLP) (yes/no), renal lithiasis (yes/no), recurrent urinary tract infection (UTI) (yes/no), HIV diagnosis time (<5 years/≥5 years), time of use of ART (<5 years/≥5 years), use of nephrotoxic anti-retrovirals in previous therapeutic regimens (none/one/two or more), nephrotoxic anti-retrovirals in current therapeutic regimens (none/one/two or more), nephrotoxic anti-retrovirals in previous and current therapeutic regimens (none/one/two or more), hepatitis B and C co-infection (yes/no) and the last three results and laboratory tests for serum creatinine.

Age was dichotomized for statistical analysis, so greater or equal to 40 years old or below 40 years old were chosen. To this end, it was based on international articles on this theme, in which the age group older than 40 years old presented the highest risk of changes in the l function^(^ 23 ^-^ 24 ^).^

For the assessment of dyslipidemia, the last three recorded laboratory tests of blood levels of High Density Lipoproteins-Cholesterol (HDL-c,) Low Density Lipoproteins-Cholesterol (LDL-c), Total Cholesterol (TC), and Triglycerides (TGs) were considered. The registration of lipid-lowering drugs was also considered. The reference values of the Brazilian Guideline for Dyslipidemias and Atherosclerosis Prevention of the Brazilian Society of Cardiology were used(25), namely: Desirable TC <200 mg/dl; Optimum LDL-c <100 mg/dl; Desirable HDL-c >40 mg/dl; and desirable TG <150 mg/dl.

The SAH, DM, CVD, renal lithiasis, recurrent UTI, and hepatitis B and C co-infection variables were considered according to the disease report in the patients’ medical records.

As for the nephrotoxic anti-retrovirals, ARDs already proven in the literature were considered to be nephrotoxic: Tenofovir Disoproxil Fumarate (TDF)^(^ 26 ^-^ 28 ^)^, Atazanavir (ATV), Lopinavir boosted with Ritonavir (LPV/r), and Indinavir (IDV)^(^ 7 ^,^ 29 ^)^.

In order to obtain the eGFR using the CKD-EPI equation, it was first necessary to convert the conventional creatinine into Creatinine for Isotopic Dilution Mass Spectrometry (IDMS), considered the gold standard, in order to minimize its variability^(^ 30 ^)^.

CKD staging based on eGFR and albuminuria is classified into six stages (1, 2, 3A, 3B, 4 and 5); however, only stages 3A (eGFR: 45-59 mL/min/1.73 m^2^); 3B (eGFR: 30-44 mL/min/1.73 m^2^); 4 (eGFR: 15-29 mL/min/1.73 m^2^) and 5 (eGFR < 15 mL/min/1.73 m^2^)^(^ 21 ^)^ were considered, since 24-hour proteinuria tests and/or Albuminuria/Creatininuria ratio (ACR) were not analyzed in this study, it was not possible to identify CKD stages 1 and 2.

The data were processed and analyzed using the Statistical Package for Social Sciences (SPSS) software, version 25.0. Descriptive statistics were used through: absolute (n) and relative (%), mean, standard deviation (SD), minimum and maximum, in addition to Pearson›s Chi-square association tests (Χ2) and Fisher’s exact test.

The independent variables, previously associated, were submitted to logistic regression analysis. For the logistic regression, the selection of the independent variables was performed (gender, age group, smoking, hepatitis B and C, Systemic Arterial Hypertension, Diabetes Mellitus, Cardiovascular Disease, dyslipidemia, renal lithiasis, urinary tract infection, time of HIV diagnosis, time of use of ART, and current and previous nephrotoxic anti-retrovirals) through the Likelihood Ratio test. A p value<0.05 was adopted. From the chosen model, the respective gross and adjusted Odds Ratios (ORs) of the model parameters were calculated with the respective 95% Confidence Interval (CI). In all the analyses, the level of significance adopted was 5% (alpha=0.05). The program used was R (R Core Team, 2018) version 3.5.1(31).

The study was conducted in accordance with the legal and ethical standards of Resolution 466/2012. The study was approved by the Research Ethics Committee of the Ribeirão Preto Nursing School at the University of São Paulo under protocol No. 2439.373. Under CAAE No.: 79881717.8.0000.5393, approved on December 14th, 2017.

## Results

Of the 258 records analyzed initially, 6.5% (n=17) met the CKD criteria pre-established in the study. They were classified as case. After analyzing the data, it was identified that, of the 85 study participants, 17 were cases and 68 controls.

Among the participants included in the cases, the majority, 16 (94.11%), were over 40 years old; of these, 82.35% were over 50 years old, 9 (52.94%) were male, 10 (58.82%) were white-skinned and 4 (23.52%) were brown-skinned. Regarding the participants included in the controls, the majority, 45 (66.17%), were in the age group above 40 years old, and the predominant gender was male with 47 (69.11%). White and brown skin colors had the same frequency: 24 (35.29%).

Table 1 shows a higher mean age among the cases, 61.4 years old (SD=9.3) compared to the controls, 49.9 years old (SD=10.8), with the opposite occurring with the time of HIV diagnosis, use of total, current and previous ART, which were lower.

**Table 1:** Distribution of means, standard deviations, minimum and maximum age, time since HIV diagnosis, and time on anti-retroviral therapy between cases (n=17) and controls (n=68). Ribeirão Preto, SP, Brazil, 2018

Variables	Cases			Controls	
	Mean (SD*)	Minimum-Maximum		Mean(SD*)	Minimum-Maximum
Age	61.4 (9.3)	46-74.6		49.9 (10.8)	21.1-72.3
Time of HIV diagnosis	13.2 (6.5)	3.9-22.5		16.8 (6.7)	2.3-27.6
Time on ART^†^ (total)	11.9 (7.4)	3.5-21.2		14.8 (6.3)	1.6-27.0
Time on ART^†^ (current)	3.6 (2.9)	0.1-11.7		4.1 (3.0)	0.5-12.3
Time on ART^†^ (previous)	8.3 (5.3)	0.8-17.0		9.8 (7.4)	0-22.4

* SD = Standard Deviation;

† ART =Anti-Retroviral Therapy

The distribution of the last three eGFR of the cases and controls according to the CKD stages is shown in Table 2. A progression in the reduction of eGFR was identified between the last three assessments in the individuals selected as case, whereas the controls presented mostly eGFR≥90 ml/min/1.73m2 and eGFR 60-89 ml/min/1.73 m2.

**Table 2:** Distribution of the last three estimates of the glomerular filtration rate of cases (n=17) and controls (n=68). Ribeirão Preto, SP, Brazil, 2018

CKD* stages	GFR^†^	Case/Control					
		**n_1_^‡^ (%)**		**n_2_^‡^ (%)**		**n_3_^‡^(%)**	
**1**	**≥90**	1(5.8)	46(67.6)	1(5.8)	49(72.0)	-	51(75.0)
**2**	**60-89**	2(11.7)	22(32.4)	-	19(28.0)	1(5.8)	16 (23.5)
**3A**	**45-59**	7 (41.1)	-	11(64.7)	-	87(47.0)	1 (1.4)
**3B**	**30-44**	7(41.1)	-	4 (23.5)	-	4(23.5)	-
**4**	**15-29**	-	-	1(5.8)	-	4 (23.5)	-
**5**	**-**	-	-	-	-	-	-
	**Total**	17(100)	68(100)	17(100)	68(100)	17(100)	68(100)

* CKD = Chronic Kidney Disease;

† GFR=Glomerular Filtration Rate measured in ml/min/1.73 m2; ‡n1, ‡n2 and ‡n3 = Correspond respectively to the ante-penultimate, penultimate, and last number of people distributed in each GFR range of cases and controls

Table 3 shows the distribution of the use of current, previous nephrotoxic anti-retroviral drugs and in both therapeutic regimens. It was identified that, among the cases, there was an increase in the use of only one nephrotoxic ARD (11.7% to 82.3%) and a reduction in the use of two or more (52.9% to 17.6%). A higher frequency of nephrotoxic ARDs was also observed in the previous and current therapeutic regimens among the cases (64.7%) whereas, in the controls only 35.2% used nephrotoxic ARDs in both therapeutic regimens.

**Table 3:** Distribution of the use of current and previous nephrotoxic anti-retrovirals among cases (n=17) and controls (n=68). Ribeirão Preto, SP, Brazil, 2018

Nephrotoxic anti-retrovirals	Case (n=17)		Control (n=68)	
	**n**	**%**	**n**	**%**
Current use*				
None	00	00	04	5.8
One	14	82.3	36	52.9
Two or more	03	17.6	28	41.1
Previous use^†^				
None	06	35.2	44	64.7
One	02	11.7	06	8.8
Two or more	09	52.9	18	26.4
Previous and current use^‡^				
None	00	00	01	1.4
In both	11	64.7	24	35.2

* Current use of one or more of a nephrotoxic anti-retroviral;

† Previous use of one or more of a nephrotoxic anti-retroviral;

‡ Concomitant use of nephrotoxic anti-retrovirals

Tables 4 and 5 show, respectively, the description and logistic regression of the variables considered as association factors (gender, age, smoking, hepatitis B and C) and (comorbidities, HIV-related clinical condition and the use of ART) to develop CKD between cases and controls. Age, SAH, and nephrotoxic anti-retrovirals in a previous treatment regimen showed a statistically significant association with CKD in this study. Age below 40 years old is therefore a protective factor (OR=0.122, p=0.022) and the diagnosis of SAH (OR=5.8, p=0.001) and previous use of nephrotoxic ARDs (OR=3.3 , p=0.028) are associated factors.

**Table 4:** Analysis of the associated factors (gender, age group, smoking, comorbidities) between cases (n=17) and controls (n=68) in people living with HIV. Ribeirão Preto, SP, Brazil, 2018

Associated factors		Case	Control	Χ^2^ *	p value^†^	Gross OR^‡^	Adjusted OR^‡^	CI^§^ (95%)
**Gender**	Male	9	47	1.58	0.200	0.56	0.39	0.17-18.5
	Female	8	21					
**Age group**	<40 years old	1	23	5.240	**0.022**	0.12	11.42	0.015-0.981
	>40 years old	16	45					
**Smoking**	Yes	4	19	0.187	0.666	0.76	1.70	0.22-2.632
	No	13	47					
**Hepatitis B**	Yes	0	3	0.777	1.0^║^	0.79	NE^¶^	0.71-0.885
	No	17	65					
**Hepatitis C**	Yes	0	62	1.614	0.342^║^	0.78	NE^¶^	0.699-0.881
	No	17	6					
**Systemic Arterial Hypertension**	Yes	9	11	10.21	**0.001**	5.83	8.37	1.84-18.42
	No	8	57					
**Diabetes Mellitus**	Yes	3	4	2.49	0.115	3.42	1.35	0.68-17.06
	No	14	64					
**Cardiovascular diseases**	Yes	3	3	3.375	0.066	4.42	0.29	0.807-24.29
	No	14	62					
**Dyslipidemia**	Yes	15	54	0.53	0.463	0.61	8.82	0.157-2.403
	No	2	13					
**Renal lithiasis**	Yes	2	4	0.717	0.397	2.13	11.78	0.357-12.75
	No	15	64					
**Urinary tract infection**	Yes	1	5	0.045	0.832	0.78	0.68	0.086-7.22
	No	16	63					

* X^2^= Chi-square;

† p value = Significance value;

‡ OR = Odds Ratio;

§ CI = Confidence Interval; ║ = Fisher’s Exact test.

¶ NE = Not Estimated (the corresponding parameter was not estimated by the model due to the occurrence of numerical problems)

**Table 5:** Analysis of the associated factors (clinical conditions related to HIV and to the use of anti-retroviral therapy) between cases (n=17) and controls (n=68). Ribeirão Preto, SP, Brazil, 2018

Associated factors		Case	Control	Χ^2^ *	p value^†^	Gross OR^‡^	Adjusted OR^‡^	CI^§^ (95%)
Time of HIV diagnosis	<5 years	5	19	0.015	0.904	1.07	1.18	0.333-3.462
	≥5 years	12	49					
Time of anti-retroviral therapy use	<5 years	8	28	0.087	0.768	1.17	0.97	0.402-3.429
	≥5 years	9	37					
Viral load^║^	Undetectable	15	62	0.329	0.567	0.60	0.44	0.107-3.426
	Detectable	2	5					
Nephrotoxic anti-retrovirals (current) ^¶^	Yes	17	64	1.049	0.306**	0.79	NE^††^	0.706-0.884
	No	0	4					
Nephrotoxic anti-retrovirals (previous) ^‡‡^	Yes	11	24	4.857	0.028	3.36	446099.48	1.105-10.221
	No	6	44					

* X^2^= Chi square;

† p value = Level of significance;

‡ OR = Odds Ratio;

§ CI = Confidence Interval;

║ Last viral load test;

¶ Use of anti-retrovirals in current therapeutic regimen;

** Fisher’s exact test;

†† NE = Not Estimated (the corresponding parameter was not estimated by the model due to the occurrence of numerical problems);

‡‡ Use of anti-retrovirals in a previous therapeutic regimen

## Discussion

Among the 17 cases and 68 controls analyzed in this study, we found that the age, SAH, and nephrotoxic anti-retrovirals in a previous treatment regimen variable showed a statistically significant association with CKD. Being less than 40 years old is a protective factor and, as associated factors, having SAH and making previous use of nephrotoxic ARDs are noted.

The main findings of this study indicate the importance of detecting the associated factors for traditional CKD, those intrinsically related to the fact of living with HIV and being treated with potentially nephrotoxic anti-retrovirals. International studies have addressed this issue in recent times due to its relevance in the population living with HIV^(^ 13 ^,^ 23 ^,^ 28 ^,^ 32 ^)^.

Chronic Kidney Disease has a low incidence of PLHIV, although it is considered an important worldwide public health problem and is more prevalent among PLHIV than in the general population^(^ 33 ^-^ 34 ^)^. This fact justifies the need to investigate the main predictors for CKD in PLHIV using the case-control methodology. This study, therefore, is the first that addresses this type of design in this theme at the national level.

From the pre-established criteria for the definition of CKD, we found a prevalence of 6.5%, which is equivalent to the cases described in this study. Among the 17 cases, stages 3A, 3B and 4 are found, with PLHIV not being identified in stage 5- Terminal Chronic Kidney Disease. This result characterizes the cases of this study. The prevalence found confirms the result obtained in a prospective multi-center European cohort study in which it followed PLHIV between the years 2006 and 2014, in which an increase from 4.1% to 6.9% of CKD^(^ 32 ^)^ was observed.

A wide variation in the prevalence of CKD is found in different locations, which is justified by the heterogeneous population, regional, cultural, structures of access to health services and differences in diagnostic criteria^(^ 13 ^,^ 23 ^,^ 28 ^)^. Based on the first systematic review that provided prevalence estimates for CKD in PLHIV in various regions of the World Health Organization, using the CKD-EPI equation, an overall prevalence of 4.8% was identified (CI: 2.9-7.1), with the highest prevalence found in Africa and the lowest in Europe^(^ 13 ^)^.

In studies that used the CKD-EPI equation and did not evaluate the presence of proteinuria, the variation comprised between 3% (Texas), 3.3% (Brazil), 5% (Australia), 5.4% (London) and 13.4% (Africa), among people living with HIV^(^ 18 ^,^ 27 ^,^ 35 ^-^ 37 ^)^.

While those who analyzed GFR<60 ml/min/1.73 m^2^ and presence of proteinuria in more than one measure, in a recent study showed a global presence of 11.7% of CKD, with 7.6% being diagnosed only by the proteinuria criterion^(^ 38 ^)^. As well as what is represented in the Dutch (18.3%)^(^ 28 ^)^ and Vietnamese cohorts (8.3%)^(^ 34 ^)^. Data which show a high prevalence of the disease when using the two diagnostic criteria.

Being under the age of 40 has been shown to be a protective factor for CKD, that is, PLHIV under the age of 40 are less likely to develop CKD than those over 40 years old. This corroborates with other researchers who point out that, with increasing age there is a greater predisposition to lower eGFR, to greater regression of renal function and, consequently, to develop CKD^(^ 23 ^-^ 24 ^)^.

Among the non-modifiable associated factors for developing CKD, advanced age is considered an independent marker of decline in renal function in PLHIV by several scholars^(^ 18 ^,^ 27 ^-^ 28 ^,^ 38 ^-^ 39 ^)^. The same is also identified in this study, in which the cases have a mean age of 61.4 years old and, in the controls, 49.9 years old.

The relationship between increasing age and CKD was also studied in a multi-center cohort carried out over nine years with 23,000 PLHIV, in which an increasing prevalence of CKD was identified with the advancing age groups in which these individuals are inserted. Among people aged between 50 and 60 years old, there was an increase from 5.2% to 7.2% while, among those over 60, there was an increase from 18.5% to 23.2% of CKD^(^ 32 ^)^.

However, the characteristic of premature aging among PLHIV causes middle-aged adults using ART to have a high inflammatory exposure and, therefore, premature onset can occur of chronic comorbidities, such as CKD^(^ 24 ^,^ 40 ^)^. This assertion is in line with the results found in a retrospective observational study over a six-year period, which revealed a reduction in the mean age among those who had CKD from 49.4 to 46.4 years old (p<0.0001)^(^ 40 ^)^.

Regarding the comorbidities identified, SAH was the only chronic disease that had a strong association with CKD. PLHIV diagnosed with SAH are 5.8 times more likely to develop CKD compared to those who do not have SAH. This finding confirms that hypertension is a significant factor and is independently associated with the rapid decline in PLHIV’s eGFR, as found in recent international cohorts^(^ 23 ^-^ 24 ^,^ 39 ^,^ 41 ^-^ 42 ^)^.

A study carried out in Spain with 1,596 PLHIV corroborates this information when considering that the absence of hypertension is an independent marker of improvement in renal function over time (OR: 0.212; 95% CI: 0.061-0.733; p<0.014)^(^ 23 ^)^. In addition, other researchers have shown a higher burden of hypertension among PLHIV when compared to the general population^(^ 42 ^-^ 45 ^)^.

Another variable that showed a significant association was the use of nephrotoxic anti-retrovirals in a previous therapeutic regimen. Individuals who used them were 3.3 times more likely to develop CKD compared to those who did not. This fact can be explained by the longer time of exposure to these specific ARDs in previous ART compared to the current one^(^ 7 ^)^.

A multi-center cohort study corroborates with this study by showing that the cumulative exposure of nephrotoxic ARDs during the first five years of follow-up shows a progression in renal impairment rates^(^ 7 ^)^. Therefore, PLHIV who had GFR>90 ml/min/1.73 m^2^ and started the ART with Tenofovir, Atazanavir, Lopinavir enhanced by Ritonavir, and Darunavir showed a significant and growing association of CKD^(^ 7 ^)^.

Due to the nephrotoxicity of TDF recognized by the literature, the Ministry of Health recommends the discontinuation of this drug in a timely manner (six months of exposure) and its replacement by another ARD if, when using this drug, there is a 25% decline in baseline eGFR or GFR<60 ml/min/1.73 m^2(^ 5 ^)^.

By postponing the discontinuation of nephrotoxic ARDs, transient damage to renal structures can become permanent^(^ 26 ^,^ 28 ^,^ 46 ^)^. Both the high prevalence of the use of nephrotoxic ARDs in a previous and current scheme, and the cumulative exposure of these ARDs may justify the number of cases found in this study.

In this context, the nurse, as a member of a health team, is a professional who articulates, coordinates and conducts care interventions through goals, interventions and, consequently, obtaining satisfactory results. Its role is fundamental for the identification of the factors associated with CKD and problem solving^(^ 47 ^)^.

In addition, the nursing team must perform health education actions in the prevention, early detection, and management of CKD in order to change behaviors and lifestyle habits^(^ 48 ^)^. Thus, providing the protagonism of care for PLHIV from the knowledge of the risks to the chronic diseases that they are exposed to, CKD being an important comorbidity that can affect these individuals.

As for the limitations of the study, although there is methodological rigor from the preparation of the study model to the analysis of the data, some should be noted due to the retrospective nature of the study. Data collection was performed exclusively from secondary data in medical records of patients and, therefore, there were some gaps in obtaining the information.

Another limitation of the study concerns the unavailability of more than one result of 24-hour proteinuria or ACR tests. In view of this, the definition for CKD depended only on the criterion of eGFR<60 ml/min/1.73 m^2^. Therefore, the identification of CKD stages 1 and 2 were impaired, whereas only the presence of albuminuria≥30 mg/24 hours or albuminuria/proteininuria ratio (APR)>30 mg/g in more than one measure for more than three months characterizes such classifications.

Such limitations did not prevent the research from being carried out, as international studies also pointed out similar difficulties with access to proteinuria tests and, therefore, used the same definition adopted in this study^(^ 18 ^,^ 27 ^,^ 36 ^)^.

## Conclusion

The factors associated with CKD in PLHIV were the presence of SAH and nephrotoxic anti-retrovirals in a previous therapeutic regimen. Another factor also associated, however, as protection for CKD in PLHIV was age below 40 years old.

The people living with HIV under study have multi-factorial exposure associated with chronic kidney disease. The presence of SAH and nephrotoxic anti-retrovirals in a previous therapeutic regimen were considered factors associated with CKD in PLHIV. On the other hand, age under 40 was considered a protective factor against CKD in PLHIV.

Thus, knowing the factors associated with CKD helps in its identification and/or in the identification of the renal dysfunction existing among PLHIV, in addition to supporting the clinical decision of the health professionals, including nurses, who directly assist them. This with a view to implementing interventions for the prevention, detection, and adequate management of CKD through frequent monitoring of renal function and existing comorbidities.

For this reason, frequent updates to the SAEs are advised on changes in the profile of PLHIV, training courses aimed at the prevention and management of CKD, construction of care protocols, as well as raising the awareness of the health professionals to interdisciplinary work and comprehensive care.
